# Comparing ant behaviour indices for fine-scale analyses

**DOI:** 10.1038/s41598-019-43313-4

**Published:** 2019-05-02

**Authors:** Patrick Krapf, Nadine Hochenegger, Wolfgang Arthofer, Birgit C. Schlick-Steiner, Florian M. Steiner

**Affiliations:** Molecular Ecology Group, Department of Ecology, University of Innsbruck, Technikerstr. 25, Innsbruck, 6020 Austria

**Keywords:** Behavioural ecology, Animal behaviour

## Abstract

Animal behaviour often is characterised by standardised assays. In social insects such as ants, behaviour assays are for example used to characterise aggressive and peaceful behaviour. Such assays differ in the number of individuals, the duration and place of assays, and the scoring scales. Also the behaviour indices used to summarise the results differ. Here, we compared five behaviour indices (Aggression Index, Mean Maximum Aggression Index; and the newly introduced Mean Maximum Peace Index, Mean Behaviour Index aggressive, and Mean Behaviour Index peaceful) using a scoring scale that comprises peaceful and aggressive behaviour. The indices were applied on eight simulations and three observed data sets. The five indices were correlated but frequently differed in their means. Multiple indices were needed to capture the complete behaviour range. Furthermore, subtle differences in workers’ behaviour, that is, differences that go beyond the presence/absence of aggression, were only identified when considering multiple indices. We infer that the indices applied are differently suited for different analyses. Fine-scale analyses of behavioural variation profit from using more than one index. The particular choice of index or indices likely influences the interpretation of behaviour and should be carefully done in the light of study species and research question.

## Introduction

Standardised behaviour assays are often used across various vertebrate and invertebrate taxa^1–4^, and especially aggression assays are widely used^5–8^. In social insects, for example ants, such behaviour assays are conducted to characterise aggressive and non-aggressive behaviour in analysing, among others, associations and symbioses^9–11^, behavioural syndromes^12–14^, competitive abilities^15,16^, colony boundaries^17–19^, and supercolonies^20–22^.

The aim and design of such behaviour assays differ notably among studies^23^. Standard behaviour assays – one-on-one encounters in a neutral arena – are performed in the field or in the laboratory^24–27^, with various numbers of individuals used^23,28^. Depending on the number of colonies and workers, the time required to collect workers and perform all encounters varies from a few days^29,30^ to several weeks or months^31,32^. Specific scoring scales are chosen to characterise the behaviour of workers. Such scoring scales frequently differ among assays, and, for example, range from 0 to 4 (ignoring, antennation, avoidance, biting antennae or legs, and fighting with both ants engaged^21^) or 0 to 5 (ignoring, antennation, avoidance, dorsal flexion, aggression, and fight; levels 0 to 2 are referred to as nonaggressive and levels 3 to 5 as aggressive behaviour^20^). Such scoring scales are sufficient to detect aggressive behaviour and are used in studies that involve frequent and pronounced aggression such as when studying neighbouring supercolonies^33^. In such studies, aggressive behaviour is better depicted than non-aggressive behaviour, which often remains underrepresented^34,35^ or is not even included in the down-stream analysis^36,37^.

Nevertheless, also peaceful behaviour is observed in some studies, which therefore use scoring scales that comprise both aggressive and peaceful behaviour^38–44^. Determining the complete behaviour range is important to detect subtle differences and variation in the individual’s behaviour. This is especially true for ant species partly or exclusively non-aggressive in intraspecific encounters (e.g., *Tetramorium alpestre*^45^, *Lasius austriacus*^46^, and 20 other ant species, references to be found in^46^). Measuring both aggressive and peaceful behaviour in such species may help to reveal and understand the eco-evolutionary development of ants. It could, for example, also help unravel how supercolonies developed, a hotly debated topic^47^.

**Figure 1:**
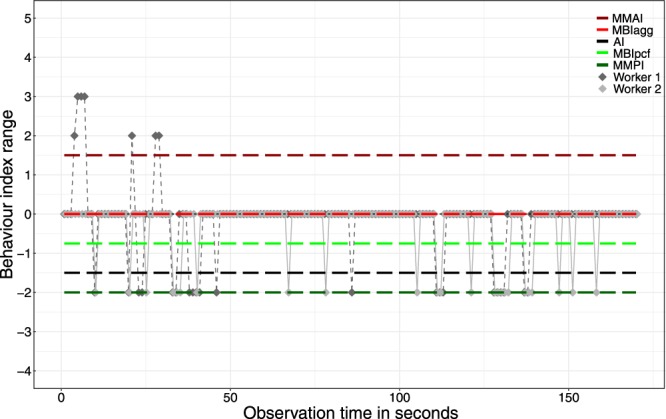
Behaviour values scored and behaviour index values calculated of an internest one-on-one encounter between *Tetramorium alpestre* workers of Data Set 2. For each worker, behaviour values are shown for each second; positive values represent aggressive behaviour, negative values peaceful behaviour (see Table 5 for details on the behaviour scale used). For the five behaviour indices Mean Maximum Aggression Index (MMAI), Mean Behaviour Index aggressive (MBI_agg_), Aggression Index (AI), Mean Behaviour Index peaceful (MBI_pcf_), and Mean Maximum Peace Index (MMPI), values of both workers were averaged. A video and an evaluation file of this encounter are available in the Supplementary Material (Video 1, Evaluation File 1).

Moreover, not only the aim, design, and scoring scales but also the behaviour indices used are diverse among studies^23,25,48–50^. Two indices are frequently analysed in behaviour assays, the Mean Maximum Aggression Index (MMAI) and the Aggression Index (AI). MMAI is frequently used in studies that essentially focus on the presence or absence of aggression^21,35–37,51^. The mean of the highest aggression values observed across the replicated encounters represents the index value. AI is frequently used in studies that focus on the frequencies of behaviour, such as when analysing nestmate recognition^34,52–54^. The duration of each behaviour is multiplied with its behaviour score and divided by the total number of seconds with tactile interaction^34^. Importantly, the values of different behaviour indices may be different when applied to the same data set. In such scenarios, a worker might get defined as aggressive using MMAI but as non-aggressive using AI as shown in Fig. 1 (for details of the analyses, see Video 1 and Evaluation File in the Supplementary Material). Obviously, such differences among behaviour indices make the results and their interpretation depend on the choice of the particular index^23^. However, how relevant such differences are in terms of their frequency and extent has remained unexplored.

The aim of this paper was to compare MMAI, AI, and three here newly introduced behaviour indices (Mean Maximum Peace Index, MMPI; Mean Behaviour Index aggressive, MBI_agg_; and Mean Behaviour Index peaceful, MBI_pcf_) in their impact on the results when applied to the same data sets in a fine-scale analysis. MMPI is the counterpart of MMAI revealing the maximum peaceful behaviour, that is, the lowest value observed. MBI_agg_ and MBI_pcf_ also reveal the maximum and minimum values, respectively, but both are calculated using time thresholds to buffer against just episodically displayed aggressive and peaceful behaviour, respectively. The thresholds thus define how long these behaviours must last so that encounters are interpreted as aggressive or peaceful (for details, see Material and Methods). Our behaviour analysis was a fine-scale analysis, in that we aimed at capturing all types of behaviour displayed. To this end, all shades of peaceful and aggressive behaviour were scored, with values from 5 to −4 and positive values representing aggression and negative values non-aggression^45^, and all behaviours were used to calculate the indices^53–57^. This approach of capturing the complete behaviour range differs from other approaches in which only aggressive behaviours were used for index calculations^13,34,38,48,58^. The behaviour indices were applied to three sets of observed data for the ant *Tetramorium alpestre* and, to cover a wider range of possible combinations of behaviours, to eight simulations, all based on one-on-one worker encounters. Specifically, we addressed three research questions: What are the advantages and disadvantages of the five behaviour indices? Is using one behaviour index sufficient to cover the complete behaviour range in fine-scale behaviour analyses, or should multiple indices be combined? If combining behaviour indices is preferable, which indices should be combined?

## Results

### Simulations and observed data sets

For both the simulations and the observed data sets, the time thresholds for MBI_agg_ and MBI_pcf_ were chosen in such a way that the contrast between aggressive and peaceful behaviour was maximised for each set of data, that is, those numbers of seconds were chosen that resulted in the highest numbers of aggressive (threshold for MBI_agg_) and peaceful (MBI_pcf_) internest encounters in a particular data set (for detailed values, see Table 1, for detailed analyses, see Materials and Methods and Supplementary Fig. S1). If not stated otherwise, mean values across all replicates and encounters of a particular set of data of behaviour indices were used throughout the analyses.

**Table 1:** Data distribution of intranest and internest encounters and highest number of aggressive and peaceful encounters and respective time thresholds used for the simulations and data sets.

Data set	Highest number of internest encounters to choose the MBIs		Time threshold [*n*] used to calculate MBIs	
	MBI_agg_	MBI_pcf_	MBI_agg_	MBI_pcf_
Data Set 1	50	18	34	3
Data Set 2	26	4	26	2
Data Set 3	13	55	47	1
Simulation P	0	10,000	169	2
Simulation P + I	0	10,000	169	2
Simulation P + I + A	4817	4782	37	68
Simulation P + I + A + K	9958	218	168	84
Simulation I + A + K	10,000	10,000	1	170
Simulation I + A	10,000	10,000	1	170
Simulation A	10,000	10,000	1	170
Simulation A + K	10,000	10,000	1	170

Note: For the eight simulations, only one distribution was calculated as no differentiation between intranest and internest was possible. *n* = observation time in seconds.

**Figure 2:**
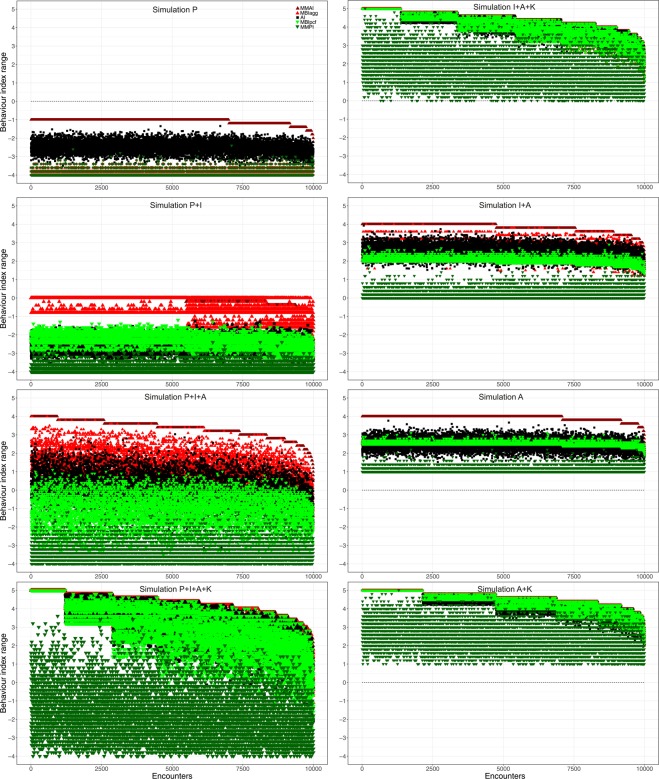
Values of the five behaviour indices Mean Maximum Aggression Index (MMAI), Mean Behaviour Index aggressive (MBI_agg_), Aggression Index (AI), Mean Behaviour Index peaceful (MBI_pcf_), and Mean Maximum Peaceful Index (MMPI) are shown for all simulations. The encounters are sorted by their MMAI values. All 10,000 encounters are displayed to reveal the complete data variability, and thus index values are partly overlaid by each other.

**Figure 3:**
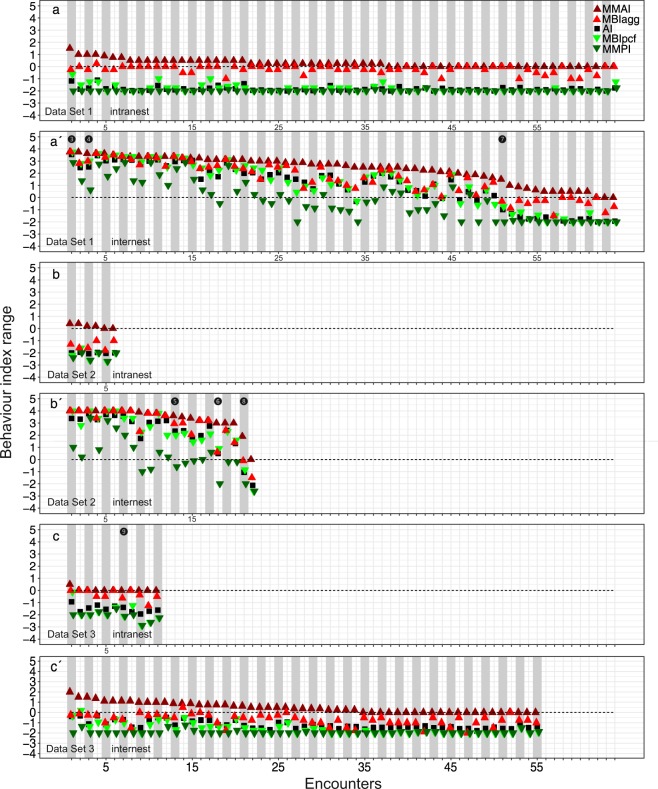
Values of the five behaviour indices Mean Maximum Aggression Index (MMAI), Mean Behaviour Index aggressive (MBI_agg_), Aggression Index (AI), Mean Behaviour Index peaceful (MBI_pcf_), and Mean Maximum Peaceful Index (MMPI) are shown for all observed data sets. The encounters are sorted by their MMAI values. Circled numbers denote examples of specific encounter types (see Fig. 4).

The eight simulations (Fig. 2) were done to explore how the behaviour indices performed when different parts of the behavioural scale (P, peaceful behaviours = −4 to −1; I, ignoring = 0; A, aggressive behaviours = 1 to 4; K, killing = 5; Table 2) were allowed to occur, in various combinations. This was done to become independent of the biology of the particular ant species, *Tetramorium alpestre*, used in the sets of observed data. The first two simulations represented intranest encounters (Fig. 2 Simulations P, P + I) and the remaining simulations represented various combinations of internest encounters (Fig. 2, Table 2). Overall, the values of the simulations and of the observed data sets displayed similar trends for the behaviour indices relative to each other (Figs 2, 3), and the observed data sets were representative examples of the behavioural variation possible in one-on-one worker encounters.

**Table 2:** Description of the simulations chosen.

Abbreviated simulations	Behaviours simulated	Scoring scales allowed	Simulations represent
Simulation P	Peaceful behaviours only	−4, −3, −2, −1	Encounters within the nest
Simulation P + I	Peaceful behaviours with ignoring	−4, −3, −2, −1, 0	Encounters within the nest
Simulation P + I + A	Peaceful and aggressive behaviours with ignoring	−4, −3, −2, −1, 0, 1, 2, 3, 4	Encounters between nests
Simulation P + I + A + K	Peaceful and aggressive behaviours with ignoring and with killing	−4, −3, −2, −1, 0, 1, 2, 3, 4, 5	Encounters between nests
Simulation I + A	Aggressive behaviours with ignoring	0, 1, 2, 3, 4	Encounters between nests
Simulation I + A + K	Aggressive behaviours with ignoring and with killing	0, 1, 2, 3, 4, 5	Encounters between nests
Simulation A	Aggressive behaviours only	1, 2, 3, 4	Encounters between nests
Simulation A + K	Aggressive behaviours with killing	1, 2, 3, 4, 5	Encounters between nests

Note: P represents peaceful behaviours, I represents ignoring, A represents aggressive behaviours, and K represents killing. Detailed information on the scoring scales used can be found in Table 5.

Looking at the individual encounters generated, throughout all simulations, some of the encounters were very similar in their values across indices, while other encounters were very different (Fig. 2). In more detail, in all simulations, MMAI and MMPI had always the highest and lowest values, respectively, among all indices (Fig. 2, see Table 3 for linear models and correlations, Table 4 for pairwise Mann-Whitney-U tests). MBI_agg_ and MBI_pcf_ values were often higher and lower than AI values, and AI values were frequently in between MBI_agg_ and MBI_pcf_ values. The highest values were detected in the simulation with aggression and killing (Fig. 2 Simulation A + K), while the lowest values were observed in the simulation with only peaceful behaviours (Fig. 2 Simulation P). The highest contrast between values was observed in the simulation with all behaviours allowed except killing (Fig. 2, Simulation P + I + A). In simulations with killing allowed or with only aggressive or only peaceful behaviour, the values of the various indices were often more similar (Fig. 2 Simulations P, P + I + A + K, I + A + K, A, A + K). Linear mixed models revealed that the values of the various behaviour indices were often positively correlated (Table 3), but the mean values were significantly different from each other as revealed by Mann-Whitney-U tests (Table 4).

**Table 3:** Pairwise linear (mixed) models and respective p-values of all behaviour indices for all data sets.

Behaviour indices	Data Set 1 intranest				Data Set 1 internest			
	MMAI	MBI_agg_	AI	MBI_pcf_	MMAI	MBI_agg_	AI	MBI_pcf_
MBI_agg_	rho = 0.23, *p* = 0.106				rho = 0.93, *p* = 0.016*			
AI	rho = 0.30, *p* = 0.082	rho = −0.21, *p* = 0.733			rho = 0.95, *p* = 0.004*	rho = 0.96, *p* = <0.001*		
MBI_pcf_	rho = 0.55, *p* = 0.075	rho = 0.09, *p* = 0.154	rho = 0.50, *p* = 0.001*		rho = 0.94, *p* = 0.006*	rho = 0.95, *p* = 0.001*	rho = 0.97, *p* = < 0.001*	
MMPI	rho = −0.07, *p* = 0.490	rho = −0.16, *p* = 0.376	rho = 0.30, *p* = 0.268	rho = 0.31, *p* = 0.244	rho = 0.86, *p* = 0.061	rho = 0.90, *p* = 0.005*	rho = 0.92, *p* = 0.016*	rho = 0.95, *p* = 0.005*
	**Data Set 2 intranest**				**Data Set 2 internest**			
MBI_agg_	rho = 0, p = 0.901				rho = 0.89, *p* = 0.029*			
AI	rho = 0.36, p = 0.430	rho = 0.44, p = 0.437			rho = 0.87, *p* = 0.008*	rho = 0.94, *p* = <0.001*		
MBI_pcf_	rho = 0.42, p = 0.325	rho = 0.54, p = 0.360	rho = 0.93, p = 0.007*		rho = 0.88, *p* = 0.005*	rho = 0.89, *p* = 0.010*	rho = 0.88, *p* = 0.018*	*
MMPI	rho = 0.25, p = 0.793	rho = 0.75, p = 0.115	rho = 0.88, p = 0.026	rho = 0.95, p = 0.009*	rho = 0.78, *p* = 0.078	rho = 0.86, *p* = 0.031*	rho = 0.90, *p* = 0.022*	rho = 0.83, *p* = 0.006*
	**Data Set 3 internest**				**Data Set 3 internest**			
MBI_agg_	rho = 0.32, *p* = 0.394				rho = 0.48, *p* = 0.002*			
AI	rho = 0.50, *p* = 0.028	rho = 0.02, *p* = 0.496			rho = 0.70, *p* = <0.001*	rho = 0.34, *p* = 0.019*		
MBI_pcf_	rho = 0.51, *p* = 0.006*	rho = 0.64, *p* = 0.055	rho = 0.57, *p* = 0.008*		rho = 0.78, *p* = 0.001*	rho = 0.49, *p* = 0.012*	rho = 0.83, *p* = <0.001	
MMPI	rho = 0.11, *p* = 0.793	rho = 0.52, *p* = 0.089	rho = 0.59, *p* = 0.036	rho = 0.82, *p* = 0.037	rho = 0.02, *p* = 0.37	rho = 0.12, *p* = 0.204	rho = 0.24, *p* = 0.008*	rho = 0.34, *p* = 0.078
	**Simulation P**				**Simulation P + I**			
MBI_agg_	rho = −0.16, *p* = <0.0020				rho = 0.77, p = <0.001*			
AI	rho = 0.22, *p* = 0.001*	rho = 0.19, *p* = <0.001*			rho = 0.17, p = <0.001*	rho = 0.01, p = 0.21		
MBI_pcf_	rho = −0.16, *p* = <0.001*	rho = 0.99, *p* = <0.001*	rho = 0.19, *p* = <0.001*		rho = 0.65, p = <0.001*	rho = 0.56, p = <0.001*	rho = 0.10, p = <0.001*	
MMPI	rho = −0.16, *p* = <0.001*	rho = 0.99, *p* = <0.001*	rho = 0.21, *p* = <0.001*	rho = 0.99, *p* = <0.001*	rho = −0.08, p = <0.001*	rho = −0.02, p = 0.359	rho = 0.24, p = <0.001*	rho = 0.31, p = <0.001*
	**Simulation P + I + A**				**Simulation P + I + A + K**			
MBI_agg_	rho = 0.42, *p* = <0.001*				rho = 0.86, *p* = <0.001*			
AI	rho = 0.30, *p* = <0.001*	rho = 0.65, *p* = <0.001*			rho = 0.83, *p* = <0.001*	rho = 0.94, *p* = <0.001*		
MBI_pcf_	rho = −0.01., *p* = 0.005*	rho = 0.51, *p* = 0.103	rho = 0.66, *p* = <0.001*		rho = 0.80, *p* = <0.001*	rho = 0.91, *p* = <0.001*	rho = 0.95, *p* = <0.001*	
MMPI	rho = −0.05, *p* = <0.001*	rho = −0.04, *p* = <0.001*	rho = 0.31, *p* = <0.001*	rho = 0.39, *p* = <0.001*	rho = 0.25, *p* = <0.001*	rho = 0.28, *p* = <0.001*	rho = 0.31, *p* = <0.001*	rho = 0.33, *p* = <0.001*
	**Simulation I + A**				**Simulation I + A + K**			
MBI_agg_	rho = 0.56, *p* = <0.001*				rho = 0.90, p = <0.001*			
AI	rho = 0.28, *p* = <0.001*	rho = 0.28, *p* = <0.001*			rho = 0.91, p = <0.001*	rho = 84, p = <0.001*		
MBI_pcf_	rho = 0.58, *p* = <0.001*	rho = 0.99, *p* = <0.001*	rho = 0.28, *p* = <0.001*		rho = 0.86, p = <0.001*	rho = 0.86, p = <0.001*	rho = 0.92, p = <0.001*	
MMPI	rho = −0.25, *p* = <0.001*	rho = 0.58, *p* = <0.001*	rho = 0.04, *p* = <0.001*	rho = 0.58, *p* = <0.001*	rho = 0.35, p = <0.001*	rho = 0.39, p = <0.001*	rho = 0.37, p = <0.001*	rho = 0.39, p = <0.001*
	**Simulation A**				**Simulation A + K**			
MBI_agg_	rho = 0.63, *p* = <0.001*				rho = 0.53, p = <0.001*			
AI	rho = 0.18, *p* = <0.001*	rho = 0.30, *p* = <0.001*			rho = 0.91, p = <0.001*	rho = 0.96, p = <0.001*		
MBI_pcf_	rho = 0.63, *p* = <0.001*	rho = 0.99, *p* = <0.001*	rho = 0.30, *p* = <0.001*		rho = 0.95, p = <0.001*	rho = 0.99, p = <0.001*	rho = 0.96, p = <0.001*	
MMPI	rho = −0.16, *p* = <0.001*	rho = 0.62, *p* = <0.001*	rho = 0.20, *p* = <0.001*	rho = 0.62, *p* = <0.001*	rho = 0.34, p = <0.001*	rho = 0.39, p = <0.001*	rho = 0.37, p = <0.001*	rho = 0.39, p = <0.001

Note: Rho values represent Spearman rank correlation values. P-values result from linear models for intranest encounters, and from linear mixed models for internest encounters and simulations. P-values were obtained using the package “lmerTest”^67^. Values with an asterisk (*) are significant after Bonferroni-Holm correction for multiple testing.

**Table 4:** Pairwise comparison of behavioural mean values and p-values of all behaviour indices for all data sets.

Behaviour indices	Data Set 1 intranest					Data Set 1 internest				
	MMAI	MBI_agg_	AI	MBI_pcf_	MMPI	MMAI	MBI_agg_	AI	MBI_pcf_	MMPI
MMAI	**0.28**					**2.35**				
MBI_agg_	<0.001*	**−0.23**				0.001*	**1.56**			
AI	<0.001*	<0.001*	**−1.84**			<0.001*	0.103	**1.00**		
MBI_pcf_	<0.001*	<0.001*	0.059	**−1.80**		<0.001*	0.175	0.766	**1.07**	
MMPI	<0.001*	<0.001*	<0.001*	<0.001*	**−1.99**	<0.001*	<0.001*	<0.001*	<0.001*	**−0.18**
	**Data Set 2 intranest**					**Data Set 2 internest**				
MMAI	**0.20**					**3.41**				
MBI_agg_	0.005*	**−1.38**				0.245	**2.83**			
AI	0.005*	0.005*	**−2.01**			<0.001*	0.072	**2.32**		
MBI_pcf_	0.005*	0.005*	0.468	**2.18**		0.006*	0.152	0.972	**2.29**	
MMPI	0.005*	0.005*	0.254	0.803	**−2.28**	<0.001*	<0.001*	<0.001*	<0.001*	**0.26**
	**Data Set 3 intranest**					**Data Set 3 internest**				
MMAI	**0.05**					**0.49**				
MBI_agg_	0.005*	**−0.34**				<0.001*	**−0.72**			
AI	<0.001*	<0.001*	**−1.51**			<0.001*	<0.001*	**1.23**		
MBI_pcf_	<0.001*	<0.001*	0.035	**−1.88**		<0.001*	<0.001*	<0.001*	**−1.58**	
MMPI	<0.001*	<0.001*	<0.001*	0.683	**−2.10**	<0.001*	<0.001*	<0.001*	<0.001*	**−1.93**
	**Simulation P**					**Simulation P + I**				
MMAI	**−1.08**					**−0.14**				
MBI_agg_	<0.001*	**−3.92**				<0.001*	**−3.86**			
AI	<0.001*	<0.001*	**−2.50**			<0.001*	<0.001*	**−2.50**		
MBI_pcf_	<0.001*	1.00	<0.001*	**−3.92**		<0.001*	1.00	<0.001*	**−3.86**	
MMPI	<0.001*	1.00	<0.001*	1	**−3.92**	<0.001*	1.00	<0.001*	1.00	**−3.86**
	**Simulation P + I + A**					**Simulation P + I + A + K**				
MMAI	**3.35**					**4.32**				
MBI_agg_	<0.001*	**1.12**				<0.001*	**3.56**			
AI	<0.001*	<0.001*	**0.01**			<0.001*	<0.001*	**3.2**		
MBI_pcf_	<0.001*	<0.001*	<0.001*	**−1.24**		<0.001*	<0.001*	<0.001*	**2.91**	
MMPI	<0.001*	<0.001*	<0.001*	<0.001*	**−3.35**	<0.001*	<0.001*	<0.001*	<0.001*	**1.78**
	**Simulation I + A**					**Simulation I + A + K**				
MMAI	**3.81**					**4.47**				
MBI_agg_	<0.001*	**2.00**				<0.001*	**4.02**			
AI	<0.001*	<0.001*	**2.49**			<0.001*	<0.001*	**4.17**		
MBI_pcf_	<0.001*	<0.001*	<0.001*	**2.00**		<0.001*	1.00	1.00	**4.01**	
MMPI	<0.001*	<0.001*	<0.001*	<0.001*	**0.19**	<0.001*	<0.001*	<0.001	<0.001	**1.64**
	**Simulation A**					**Simulation A + K**				
MMAI	**3.92**					**4.61**				
MBI_agg_	<0.001*	**2.50**				<0.001*	**4.34**			
AI	<0.001*	1.00	**2.50**			<0.001*	1.00	**4.34**		
MBI_pcf_	<0.001*	1.00	1.00	**2.50**		<0.001*	1.00	1.00	**4.34**	
MMPI	<0.001*	<0.001*	<0.001*	<0.001*	**1.08**	<0.001*	<0.001*	<0.001*	<0.001*	**2.42**

Note: Bold values represent the mean values, the remaining values represent the p-values for the pairwise comparisons of the mean values (Mann-Whitney-U). Values with an asterisk (*) are significant after Bonferroni-Holm correction for multiple testing.

In the three observed data sets, MMAI and MMPI values were also the highest and lowest, respectively, both in intranest and internest encounters (Fig. 3, Tables 3, 4). Depending on the data set, MBI_agg_ and MBI_pcf_ values were sometimes higher and lower than AI, respectively (Fig. 3); sometimes, however, the values of all three indices were similar (Fig. 3). The most aggressive and peaceful behaviour were identified in internest encounters of Data Sets 2 and 3, respectively (Fig. 3b′,c′). Almost no aggressive behaviour was detected in intranest encounters, and only MMAI and MBI_agg_ captured it (Fig. 3 a–c). Linear mixed models revealed that the behaviour index values of internest encounters were very often correlated and those of intranest encounters less frequently (Table 3). For both intranest and internest encounters, the mean index values were often significantly different (Table 4).

Like in the simulations, also in the observed data sets, the values of the various behaviour indices were sometimes different and sometimes similar across encounters (Fig. 3): On the one hand, the values of MMAI and MMPI frequently contrasted (e.g., Fig. 3b′, encounter 13). On the other hand, almost identical behaviour index values were observed, when, for example, all indices identified aggression (e.g., Fig. 3a′, encounter 1).

Overall, in all simulations with only peaceful behaviours or only aggressive behaviours including killing, the behaviour index values were often similar (e.g., Fig. 2 Simulations P, P + I, I + A + K, A + K). In some simulations, AI and MBIs revealed almost identical results and MMAI and MMPI were more different (Fig. 2 Simulations I + A, A). In contrast, in simulations with many behaviours allowed and in observed data sets, the behaviour indices differed more often than they were similar, and, although being correlated, some values were clearly separated (e.g., Fig. 2 Simulations P + I + A, P + I + A + K, Fig. 3a′, encounter 27).

## Discussion

Ant behaviour assays are diverse in their aim and study design^23^. Moreover, behaviour indices differ notably among studies, and a systematic comparison of behaviour indices has, to our knowledge, only been conducted by Roulston *et al*.^23^. In their seminal study, Roulston *et al*. used *Linepithema humile* and compared four types of aggression assays and three behaviour indices with a scoring scale ranging from 0 to 5. They revealed that within assays, behaviour indices yielded highly similar results but that assays differed greatly in their consistency among replicates. Roulston *et al*. used ants that displayed a very clear dichotomy in their behaviour when confronted with ants from another nest – either clear-cut non-aggression or clear-cut aggression. In this study, we used ants with a greater variability in their behaviour, and we therefore were interested in a more fine-scale analysis focusing on more subtle variation in the behaviour classification. We used five behaviour indices (MMAI, MMPI, AI, MBI_agg_, and MBI_pcf_) to analyse eight simulations and three observed data sets, finely resolving both aggressive and peaceful behaviour in our scoring. Overall, the index values were often significantly correlated, but their means were clearly separated from each other. This indicates that the variation in the behaviour depends on the index chosen and that different indices differently depict behavioural variation, which in turn influences the interpretation of behaviours.

All five behaviour indices revealed both advantages and disadvantages. The advantages of AI are that it is calculated easily and that it is an averaged value. By calculating the mean, AI shields from extreme, possibly non-representative values. This, however, can also be a disadvantage: When aggressive and peaceful interactions are observed equally often, they cancel each other out leading to little informative values (e.g., AI remains at or close to 0). The two behaviours do occur similarly often in the same encounter occasionally, and the influence on the results should be kept in mind; with the observed data presented here, in 2–5% of all pairings, the accumulated numbers of seconds of aggressive and of peaceful interactions differed by less than 10 seconds (DS1 4%, 39 of 1088 encounters; DS2 2%, 4 of 205 encounters; DS3 5%, 25 of 528 encounters). The advantages of MMAI and MMPI are that they likewise are calculated easily (even more easily than AI, in fact) and that they identify the most extreme aggressive and peaceful behaviours, respectively. They thus can reveal behaviours that are sometimes not identified by calculating AI. Obviously, representing extremes is also their disadvantage: They are prone to inflation by episodic brief interactions (Figs. 2, 3). The advantage of MBI_agg_ and MBI_pcf_ is that they identify extreme aggressive and peaceful behaviour values, respectively, similarly to MMAI and MMPI, but MBI_agg_ and MBI_pcf_ are based on two time thresholds, which prevent using the aforementioned extreme values observed only episodically. The thresholds define how long a behaviour must last for encounters to be interpreted as aggressive or peaceful. The particular threshold used can be chosen to adjust it to the specific needs of a research question and study organism (here, we used it in such a way, that the contrast was maximised; see results, first paragraph). Another advantage is that in calculating MBI, aggressive and peaceful behaviour cannot cancel each other out. One disadvantage is the more demanding analysis compared with AI, MMAI, and MMPI (see Index calculator in the Supplementary Material). An additional property to be noted is that MBI_agg_ and MBI_pcf_ reveal similar results when just aggressive or just peaceful behaviours occur, that is, one of them becomes redundant.

**Figure 4:**
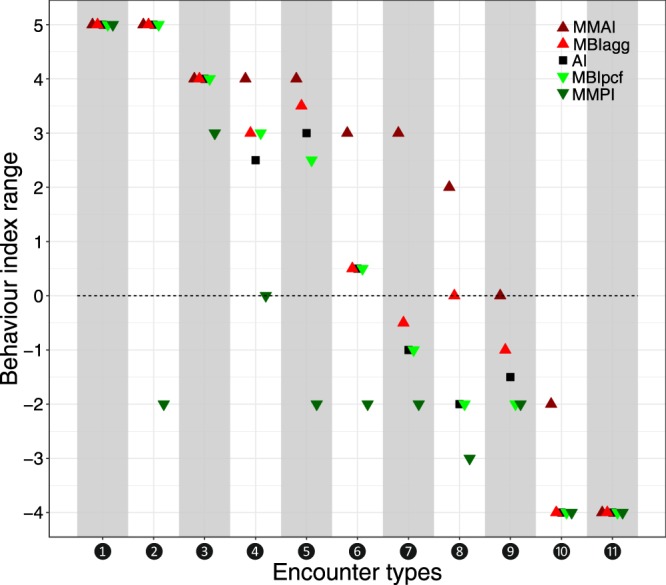
Eleven encounter types seen in simulations and observed data sets (Figs. 3 and 4) characterised by the five behaviour indices Mean Maximum Aggression Index (MMAI), Mean Behaviour Index aggressive (MBI_agg_), Aggression Index (AI), Mean Behaviour Index peaceful (MBI_pcf_), and Mean Maximum Peaceful Index (MMPI). This non-exhaustive collection of encounter types showcases that for characterising some encounter types (❶, ❷, ❿, ⓫; from simulations, not in observed data in this study), any index can be used but that for all others, the combination of multiple indices helps capturing behavioural complexity. Encounter type ❶ represents instant killing; ❷ killing after brief peaceful interactions; ❸ prolonged aggression; ❹ prolonged aggression alongside brief peaceful interactions; ❺ prolonged aggressive behaviour with brief peaceful behaviour; ❻ brief aggressive behaviour, brief peaceful behaviour, and prolonged neutral behaviour; ❼ brief aggression and prolonged peaceful behaviour; ❽ brief aggression with prolonged peaceful interactions; ❾ peaceful behaviour alongside ignoring; ❿ continuous trophallaxis alongside brief antennation; ⓫ trophallaxis throughout.

For data sets with no or almost no behavioural variation within (and across) encounters, using few indices or even just a single index in the down-stream analysis seems sufficient, although applying all behaviour indices together may be helpful to ascertain that indeed no behavioural variation applies. Such data sets may result from using coarse behaviour assays (e.g., assessment of behaviour just every 10 seconds) or from assaying encounter types such as high aggression throughout (e.g., displayed by workers from neighbouring supercolonies^33^). In Fig. 4, we have identified four such encounter types (types ❶, ❷, ❿, ⓫).

In all other instances (exemplified by types ❸ to ❾ in Fig. 4), we argue, no single index suffices and, rather, multiple indices combined may be needed to capture the complete behavioural complexity. This is rewarding as detailed behavioural information is gained. Such information obtained from fine-scale behaviour analysis can then in the down-stream analysis be linked to specific explanatory parameters (e.g., cuticular hydrocarbons, genetic relatedness, brood retrieval, brood care, etc.), which can be a relevant basis to understand the eco-evolutionary development of ants. Further, from the similarity and dissimilarity across the indices combined, information on the constancy and variation of behaviour throughout an encounter can be extracted, respectively. To demonstrate in more detail how combining indices opens up information that stays undetected when using just a single index, one encounter type is discussed here.

Encounter type ❽ represents brief aggressive behaviour with prolonged peaceful interactions (Fig. 3b′, observed Data Set 2, encounter 21): From the strong difference of the values of the different indices, strong variation of behaviour can be deduced. MMAI returns aggressive, MBI_agg_ neutral, and the other indices return peaceful values. This indicates that both aggressive and peaceful behaviours occur but peaceful behaviour is more pronounced. Without MMAI, aggressive behaviour would not have been detected, while using only MMAI, the encounter would have been classified as aggressive. MBI_agg_ in combination with MMAI makes accessible that the highest-level behaviour (2, open mandibles) lasted shorter than the time threshold used and that no other aggressive behaviour lasted longer than it, facilitating to decide whether the brief aggression is non-representative (because, e.g., due to an experimental artefact) or particularly interesting given the long peaceful interactions. MBI_pcf_ and MMPI together offer the insight that the lowest-level behaviour (−3, allogrooming) was displayed shorter than the time threshold and that, among peaceful behaviours, −2 (antennation) was the lowest level that lasted longer than it. This was also corroborated by both AI and MBI_pcf_ as their values were similar. Furthermore, encounter type ❽ suggests that for *T. alpestre*, four indices (all except AI) should be used to capture the complete behaviour range. While this might be true for this specific encounter type in *T. alpestre*, combining other indices for fine-scale analyses might be more appropriate when using different ant species or research questions.

Besides the choice of behaviour indices, this adaptability applies also to other parameters such as the scoring scale and the inclusion or exclusion of scored behaviours in the index calculation. The latter may in particular be relevant concerning antennation. If antennation is interpreted as proxy for both nestmate recognition and peaceful information exchange between workers that have recognised each other (e.g., information on foraging locations, orientation cues, or trail conditions^59–62^), it may be included in the index calculation (e.g.,^53–57^), as also done in this study. In contrast, if antennation is mainly or exclusively seen as proxy for nestmate recognition, that is, not as peaceful behaviour, it may be excluded (e.g.,^13,34,38,48,58^). Ideally, the decision whether to include or exclude antennation in the index calculation should be based on established knowledge of the various functions of antennation – a framework we are currently lacking for ants as far as we know.

In summary, applying multiple behaviour indices to a data set can help uncover behavioural complexity. This can be especially useful when focusing on shades of peaceful behaviour or transitions between peaceful and aggressive behaviour, subtle differences in worker behaviour, or behavioural variation^38–42^. The indices newly introduced in this study have been specifically adapted to detect all behavioural shades observed in one-on-one encounters using an ant species that is partly non-aggressive in intraspecific encounters (see^46^ for other such ant species). Our study elaborates on Roulston *et al*.’s^23^ insight that different behaviour indices return different aspects of the behaviour displayed – it is therefore paramount to choose the particular behaviour index or indices as needed for the particular research question and study organism. This applies not only to ant behaviour indices but likely also to behaviour indices across many other taxa^5–8^.

## Materials and Methods

### Observed data

In generating all three sets of observed data, *Tetramorium alpestre* Steiner *et al*.^63^ was used. Data Set 1 is a time series, Data Sets 2 and 3 each captured a single point in time. Data Set 1 was recorded between June and November 2016. Approximately 400 workers of *T. alpestre* were collected from two colonies at Kuehtai (K1, K2, North Tyrol, Austria; internest distance 47 m) and Penser Joch (P1, P2, South Tyrol, Italy; internest distance 50 m; Table S1). Intranest and internest encounters were performed on the following days after collection (for details, see below): 0, 1, 2, 3, 6, 9, 12, 19, 26, 33, 47, 61, 75, 103, 133, and 159. Intranest encounters were performed for all colonies, while internest encounters comprised the combinations K1K2, P1P2, K1P1, and K2P2. The numbers of intranest and internest encounters performed were the same (n = 4 encounters per each time point after collection, adding up to 64 intranest and 64 internest encounters). The high number of intranest encounters was performed to check whether the behaviour within the nest changed due to prolonged laboratory maintenance without a queen.

The experiments for Data Set 2 were conducted in September 2017. Approximately 200 workers were collected from four colonies in Kuehtai (North Tyrol, Austria; the first two nests were 25 m apart, the latter two 18 m) and from two colonies on Penser Joch (South Tyrol, Italy; nests were 47 m apart; Table S1). Intranest encounters were performed for all colonies (n = 6 encounters), while internest encounters were performed between colonies within/across populations (n = 22 encounters).

Data Set 3 is a published behaviour test^45^ conducted in June 2011. Approximately 400 workers each were collected from 11 nests in Kuehtai (North Tyrol, Austria; nest distances ranged from 9 m to 9 km; Table S1). Intranest and internest encounters were conducted for all colonies (n = 11 encounters) and colony pairings (n = 55 encounters), respectively^45^. Further information regarding all sample locations is found in the Supplementary Material (Table S1).

### Conducting one-on-one encounters and evaluation of observed behaviour

*Tetramorium alpestre* workers were collected, transported to the laboratory at the University of Innsbruck, and kept there at room temperature in polypropylene boxes (13 × 13 cm) awaiting behaviour assays. Workers were provided with water and food (Data Sets 1 and 2: sugar water and frozen *Drosophila* flies; Data Set 3: honey) ad libitum. For intranest and internest one-on-one encounter encounters, workers from the same nest and from different nests were used, respectively. For each encounter, naïve workers were randomly selected. In five encounters, too few naïve workers were available, and workers already used in intranest encounters were used for internest encounters. If workers from intranest encounters were re-used, they could acclimatise for at least 5 minutes in their native nest before being used with a non-nestmate.

For each one-on-one encounter, two workers were transferred to a small glass vial (1.4 cm diameter). Due to the arena size, workers were forced to interact with each other. Encounters were filmed for at least 180 seconds using a high-definition camera (Handycam HDR-XR 155 or HDR-PJ810E, Sony, Tokyo, Japan, or Camcorder HC-V777, Panasonic, Osaka, Japan). The first ten seconds of each encounter after the second worker had been introduced were regarded as acclimatisation time and were excluded from further down-stream analysis. The scored encounter time lasted 170 s. The observer was not blind about the origin of the workers for data sets Data Sets 1 and 3 but was blind about it for Data Set 2. The scoring scale for Data Sets 1 and 2 was identical but was different for Data Set 3 in the original work^45^. To facilitate direct comparison of all three observed data sets, the values of Data Set 3 were here translated to the scoring scale of Data Sets 1 and 2 (see Table 5). Brief explanations for each observed behaviour and behaviour frequency boxplots (Figs. S4–S7) for Data Sets 1–3 can be found in Table 5 and in the Supplementary Material, respectively.

**Table 5:** Scoring scales of observed behaviours with detailed description for Data Sets 1–3.

Observed behaviours	Scoring scale used in Data Sets 1 and 2	Names of behaviour used in scoring Data Sets 1 and 2	Original scoring scale used in Data Set 3 in Krapf *et al*.^45^	Names of behaviour used in scoring Data Set 3	Adapted scoring scale for Data Set 3 used in this study
Killing results from lower-level behaviour. Killing reveals information on maximum determination and the superiority of one of the opponents, which both are relevant when assaying the behaviour two particular opponents engage in. Killing is absolute, in that after killing, the behaviour observation stops.	5	Killing			
Fighting with gaster flexion is biting and trying to sting (e.g., Myrmicinae) or spraying with, e.g., acid (Formicinae) the other worker.	4	Fighting with gaster flexion	7	Fighting	4
Fighting is biting without gaster flexion.	3	Fighting without gaster flexion	6	Biting	3
Mandible threatening is opening the mandibles to display potential aggressiveness but without biting the other worker.	2	Mandible threatening	5	Mandible threatening	2
Avoiding is fleeing from the other worker, usually by walking backwards.	1	Avoiding	4	Avoiding	1
Ignoring is the lack of an interaction of workers; workers can thus be both sitting, in a distance of more than 1 mm from each other, or one or both workers can be moving.	0	Ignoring	0	Ignoring	0
Being next to each other is sitting of both workers in close proximity (1 mm at the maximum) to each other, following contact.	−1	Being next to each other without touching	1	Being next to each other without touching	−1
Antennation is the touching of the other worker with the antennae. During antennation, workers assess whether the other worker belongs to the same colony (nestmate) or not (non-nestmate). Furthermore, during a prolonged antennation time, information on food sources, foraging locations, orientation cues, and trail conditions, among others, might be exchanged between workers^59–62^, that is, antennation is meaningful also in context other than nestmate recognition. Assessing antennation is important for ant species where no information on nestmate recognition has been available and for ant species which are non-aggressive in intraspecific aggression assays but which can discriminate between nestmates and non-nestmates.	−2	Antennation	2	Antennation	−2
Allogrooming is the cleaning of another worker.	−3	Allogrooming	3	Allogrooming and trophallaxis	−3
Trophallaxis is the exchange of food or fluids between workers during which also chemical cues, growth proteins, and hormones are transferred^69^.	−4	Trophallaxis			

Note: The scoring scales used in this study are based on a pilot study using worker encounters of multiple ant species (Myrmicinae, Formicinae). Various encounters were performed, and the behaviours of the workers were filmed, analysed, and categorised. See Supplementary material for a detailed description of the behaviours used and for frequency boxplots of Data Sets 1–3 (Figs. S2–S7). The scoring scale of Data Set 3 was adapted to fit the scoring scales of Data Sets 1 and 2.

Using the videos, the behaviour of each worker was scored individually for each second, and all behaviour indices were calculated afterward for each worker in each encounter. In Data Sets 1 and 3, encounters were replicated four times and in Data Set 2, five times. The values of the behaviour indices of all workers were averaged over the replicates (henceforth encounters) to obtain mean values. For details on the index calculations, see “Behaviour index calculations” below. In all data sets, values below zero were peaceful and values above aggressive.

### Data simulation and evaluation

To analyse similarities and dissimilarities among the behaviour indices, eight simulations were created to meet all possible conditions in one-on-one encounters. The simulations were generated using a custom Python script (Python Software Foundation. Python Language Reference, version 2.7. available at http://www.python.org; the Python script is available in the Supplementary Material). In all simulations, the same scoring scale was applied. All eight simulations consisted of 10,000 averaged encounters, and one averaged encounter comprised five encounters, each lasting 170 seconds. The average calculated over these five encounters represented the mean behaviour of a colony. The first two simulations represented intranest encounters with peaceful behaviour, and the other six simulations represented internest encounters with different scenarios of only aggressive behaviour or mixed behaviour (see Tables 2 and 5 for details). Since in the Simulation P + I + A + K + all behaviours were present, it is used here to describe the simulation process: First, one behaviour was randomly selected without replacement from the pool of all ten behaviours possible. Then, a duration between one and 170 seconds was randomly chosen and assigned to the behaviour, and the remaining seconds of the encounter were calculated. These two steps were repeated until one of the following stop criteria applied: (i) All 170 seconds were assigned to behaviours and the remaining behaviours were assigned zero seconds; (ii) only one behaviour was left, to which the remaining seconds were assigned; or (iii) the behaviour “killing” was drawn and immediately assigned the remaining seconds. Thus, every single encounter simulated, for each of the 10 behaviours, all possible duration values could potentially be obtained. The same procedure as described above was applied to the remaining simulations, but zero seconds were assigned to all the behaviours not included in the simulation.

### Behaviour index calculations

In the downstream analysis, the following five indices were used. (1) The Mean Maximum Aggression Index, MMAI, was the highest aggressive score of each encounter averaged across replicate encounters of each colony pair^20,21,35^. (2) The here newly introduced Mean Maximum Peace Index, MMPI, is the counterpart to the MMAI. It was based on the highest peaceful score of each encounter, that is, the lowest value observed, and it was calculated in analogy to MMAI. (3) The Aggression Index, AI^34^, was calculated as follows: $AI=\frac{{\Sigma }_{i=1}^{n}A{I}_{i}\times {t}_{i}}{T}$ (1), where *AI*_*i*_ and *t*_i_ were the aggression index and duration of the *i*th behaviour, and *T* was the sum of the durations of all interactions with physical contact.

**Figure 5:**
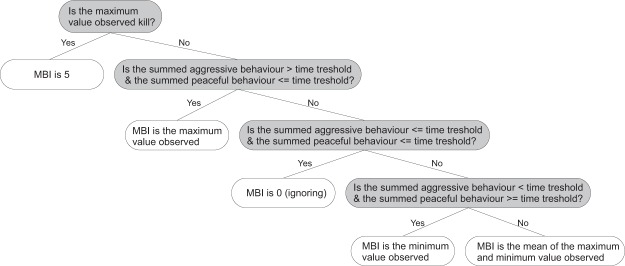
Decision cascade applied in the Mean Behaviour Index (MBI) evaluation. The decision cascade is applied for every second *n* in an encounter, that is, 170 seconds in this study (for details, see Materials and Methods). Grey and white boxes represent algorithmic queries and potential outcomes, respectively.

Here, the (4) Mean Behaviour Index aggressive (MBI_agg_) and (5) the Mean Behaviour Index peaceful (MBI_pcf_) are introduced. The Mean Behaviour Index (MBI) is a novel index that combines the sensitivity of MMAI and MMPI for brief behavioural episodes and the mitigating effects of AI. For its calculation, additionally to the second-per-second scored behaviour, a time threshold *t* is used. This time threshold can be any value between zero and the duration of the encounter in seconds (in this study, 170 s). The determination of the MBI consists of the following steps: (i) If the encounter resulted in the death of one individual, the MBI is 5. (ii) Otherwise, MMAI and MMPI are determined. (iii) The total durations of all peaceful behaviours (score < 0; in seconds) and of all aggressive behaviours (score > 0) are counted. (iv) If the duration of all aggressive behaviours is larger than *t*, but the duration of all peaceful behaviours is not, that is, aggression was the dominating behaviour during the encounter, the MBI is identical to the MMAI. (v) If both durations are smaller than *t*, that is, ignoring was the dominating behaviour, the MBI is zero. (vi) If the duration of all peaceful behaviours is larger than *t*, but that of the aggressive behaviours is not, that is, peace was the dominating behaviour, the MBI is identical to the MMPI. (vii) Finally, if the durations of both peaceful and aggressive behaviour are larger than *t*, that is, the encounter was dominated by switches between distinct aggression and distinct peacefulness, the MBI is the arithmetic mean of MMAI and MMPI (Fig. 5).

It is obvious that the value of *t* has substantial impact on the MBI: The larger *t* is, the longer a distinct behaviour (aggression, non-aggression) must have been observed to be accounted for. In the case of *t* = 0, a single second of any behaviour other than ignoring influences the MBI. In contrast, at *t* equal to the duration of the encounter, only one, uninterrupted type of behaviour throughout the whole encounter is registered. With increasing *t*, the MBI will tend towards zero (Fig. S1).

Two values of *t* are of special importance, in that they represent the *t* for MBI_agg_ and MBI_pcf_. These two values of *t* are determined empirically for each data set using only internest encounters: By calculating MBI for all possible values of *t* and plotting the number of encounters that are classified as peaceful and aggressive according to the increasing *t*, two inflection points become visible (Fig. S1, red and blue hairlines): At one particular value of *t*, the number of peaceful encounters reaches a maximum and, with further increasing *t*, stays constant or even decreases. The lowest value of *t* at which this maximum is reached, is the threshold for MBI_pcf_ (Fig. S1, blue hairline). This is done in analogy on the curve of the number of aggressive encounters with increasing *t*, resulting in the MBI_agg_ (Fig. S1, red hairline).

### Statistical analyses

All statistical analyses were performed in R v. 3.5.1 using RStudio v. 1.1.463^64,65^. All data were checked for normal distribution using the Anderson-Darling test for normality (function “ad.test”) in the R package nortest. All eight simulations and three observed data sets were analysed separately. In the observed data sets, intranest and internest encounters were also compared separately. To test whether behaviour index values were correlated, the index values of all intranest encounters were compared using linear models, while the index values of all internest encounters and of all simulations were compared using linear mixed models using the function “lmer” in the package lme4^66^. For both linear and linear mixed models, one index was set as dependent variable and the second index as independent variable. For linear mixed models, the ID of the nests and, with DS 1, the days when encounters had been performed, were set as random effects. P-values for linear mixed models were obtained using the package lmerTest^67^. Coefficients of determination were calculated for all combinations of behaviour indices in observed data sets and simulations using the Spearman rank correlation. To test whether behaviour indices yielded similar results, the mean values of all behaviour indices were compared using the Wilcoxon-Mann-Whitney-test for each simulation and set of observed data. In all statistical tests, the alpha applied was 0.05, and all values were Bonferroni-Holm corrected for multiple testing. All graphs were created using the R package “ggplot2”^68^.

## Supplementary information

Exemplary video

Supplementary data

Supplementary table and figures

## Data Availability

The datasets generated and/or analysed during the current study are available in the Supplementary Material.
