# Supplementary material for: Comparing ant behaviour indices for fine-scale analyses
Source: Sci Rep. 2019 May 2;9:6856. doi: 10.1038/s41598-019-43313-4 (PMC6497665; doi:10.1038/s41598-019-43313-4)
Supplement: Supplementary file 3 — Supplementary table and figures [file 41598_2019_43313_MOESM3_ESM.pdf]

Title

Comparing ant behaviour indices for fine-scale analyses

Patrick Krapf, Nadine Hochenegger, Wolfgang Arthofer, Birgit C. Schlick-Steiner, Florian M. Steiner
Molecular Ecology Group, Department of Ecology, University of Innsbruck, Technikerstr. 25, Innsbruck 6020, Austria

Table S1. Characteristics of the colonies assayed.

Data set	ID	Location	Country	Species	Latitude	Longitude
**Data Set 1**	K1	Kuehtai	Austria	*T. alpestre*	47.21923 N	11.07521 E
**Data Set 1**	K2	Kuehtai	Austria	*T. alpestre*	47.21929 N	11.07582 E
**Data Set 1**	P1	Penser Joch	Italy	*T. alpestre*	46.81439 N	11.44237 E
**Data Set 1**	P2	Penser Joch	Italy	*T. alpestre*	46.81404 N	11.44196 E
**Data Set 2**	18986	Kuehtai	Austria	*T. alpestre*	47.21935 N	11.07549 E
**Data Set 2**	18987	Kuehtai	Austria	*T. alpestre*	47.21925 N	11.07578 E
**Data Set 2**	18988	Kuehtai	Austria	*T. alpestre*	47.21423 N	10.99767 E
**Data Set 2**	18989	Kuehtai	Austria	*T. alpestre*	47.21407 N	10.99767 E
**Data Set 2**	18992	Penser Joch	Italy	*T. alpestre*	46.81412 N	11.44198 E
**Data Set 2**	18993	Penser Joch	Italy	*T. alpestre*	46.8144 N	11.44244 E
**Data Set 3**	17808	Kuehtai	Austria	*T. alpestre*	47.2097 N	11.1025 E
**Data Set 3**	17809	Kuehtai	Austria	*T. alpestre*	47.2192 N	11.0769 E
**Data Set 3**	17810	Kuehtai	Austria	*T. alpestre*	47.2096 N	11.1024 E
**Data Set 3**	17811	Kuehtai	Austria	*T. alpestre*	47.2099 N	11.1036 E
**Data Set 3**	17812	Kuehtai	Austria	*T. alpestre*	47.2099 N	11.1035 E
**Data Set 3**	17813	Kuehtai	Austria	*T. alpestre*	47.2097 N	11.0847 E
**Data Set 3**	17815	Kuehtai	Austria	*T. alpestre*	47.2192 N	11.0756 E
**Data Set 3**	17816	Kuehtai	Austria	*T. alpestre*	47.2192 N	11.0758 E
**Data Set 3**	17817	Kuehtai	Austria	*T. alpestre*	47.2194 N	11.0767 E
**Data Set 3**	17818	Kuehtai	Austria	*T. alpestre*	47.2208 N	10.9858 E
**Data Set 3**	17819	Kuehtai	Austria	*T. alpestre*	47.2214 N	10.9861 E

Note: ID represents a code for different nests used in behaviour assays.

Fig. S1

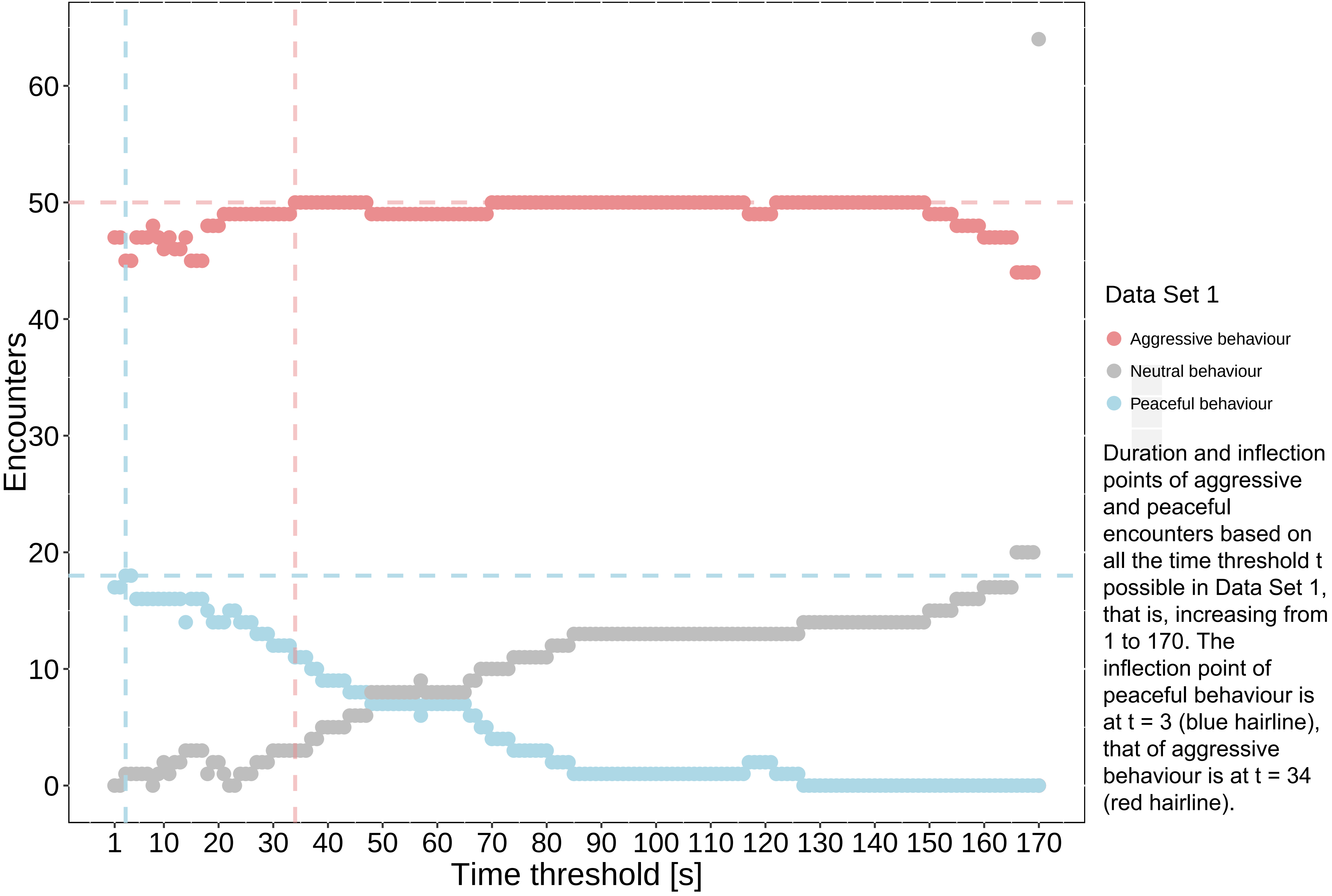

Duration and inflection points of aggressive and peaceful encounters based on all the time threshold t possible in Data Set 1, that is, increasing from 1 to 170. The inflection point of peaceful behaviour is at t = 3 (blue hairline), that of aggressive behaviour is at t = 34 (red hairline).

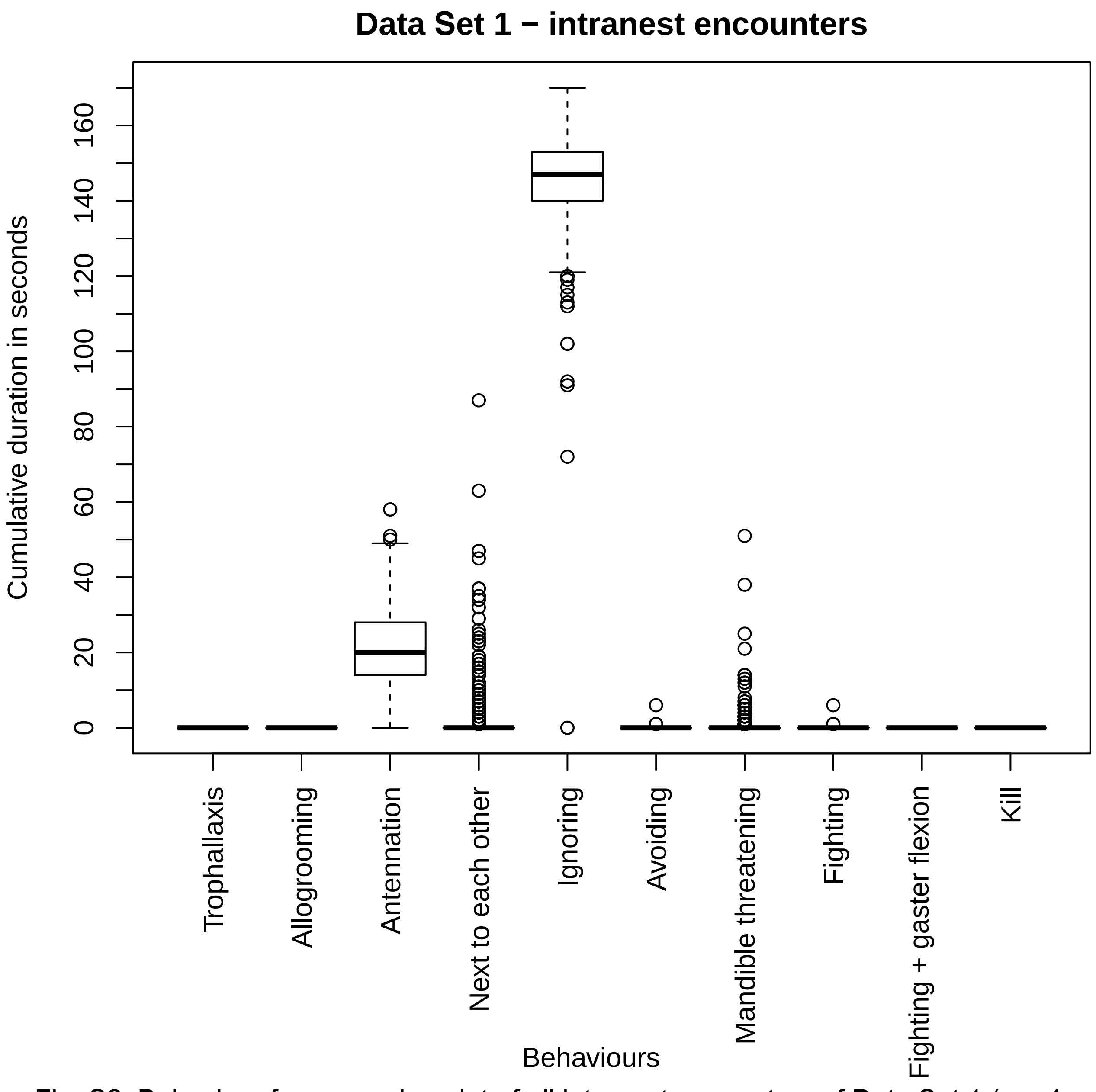

Fig. S2. Behaviour frequency boxplot of all intranest encounters of Data Set 1 (n = 4 encounters per each time point after collection, adding up to 64 intranest encounters).

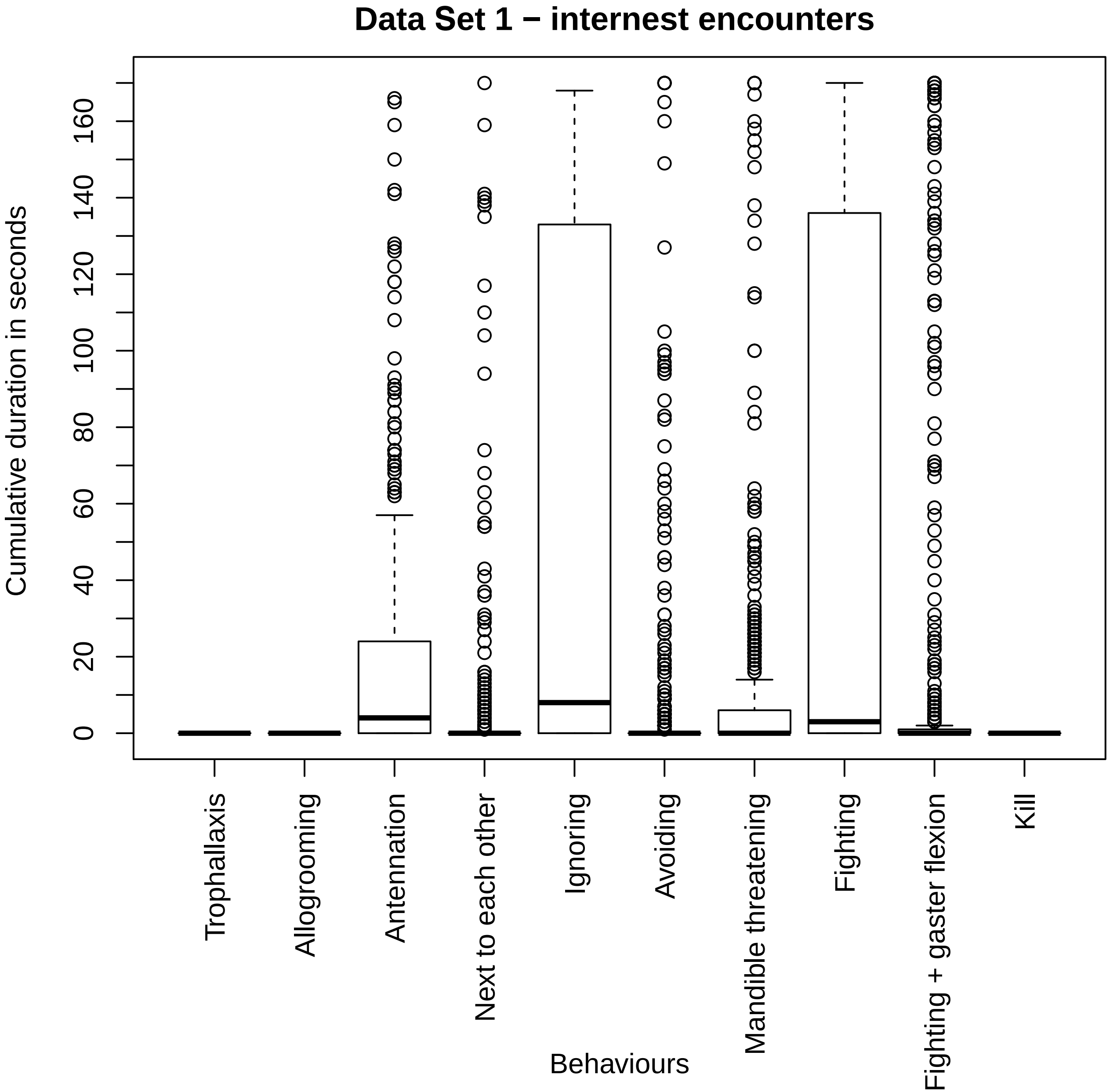

Fig. S3. Behaviour frequency boxplot of all internest encounters of Data Set 1 (n = 4 encounters per each time point after collection, adding up to 64 internest encounters).

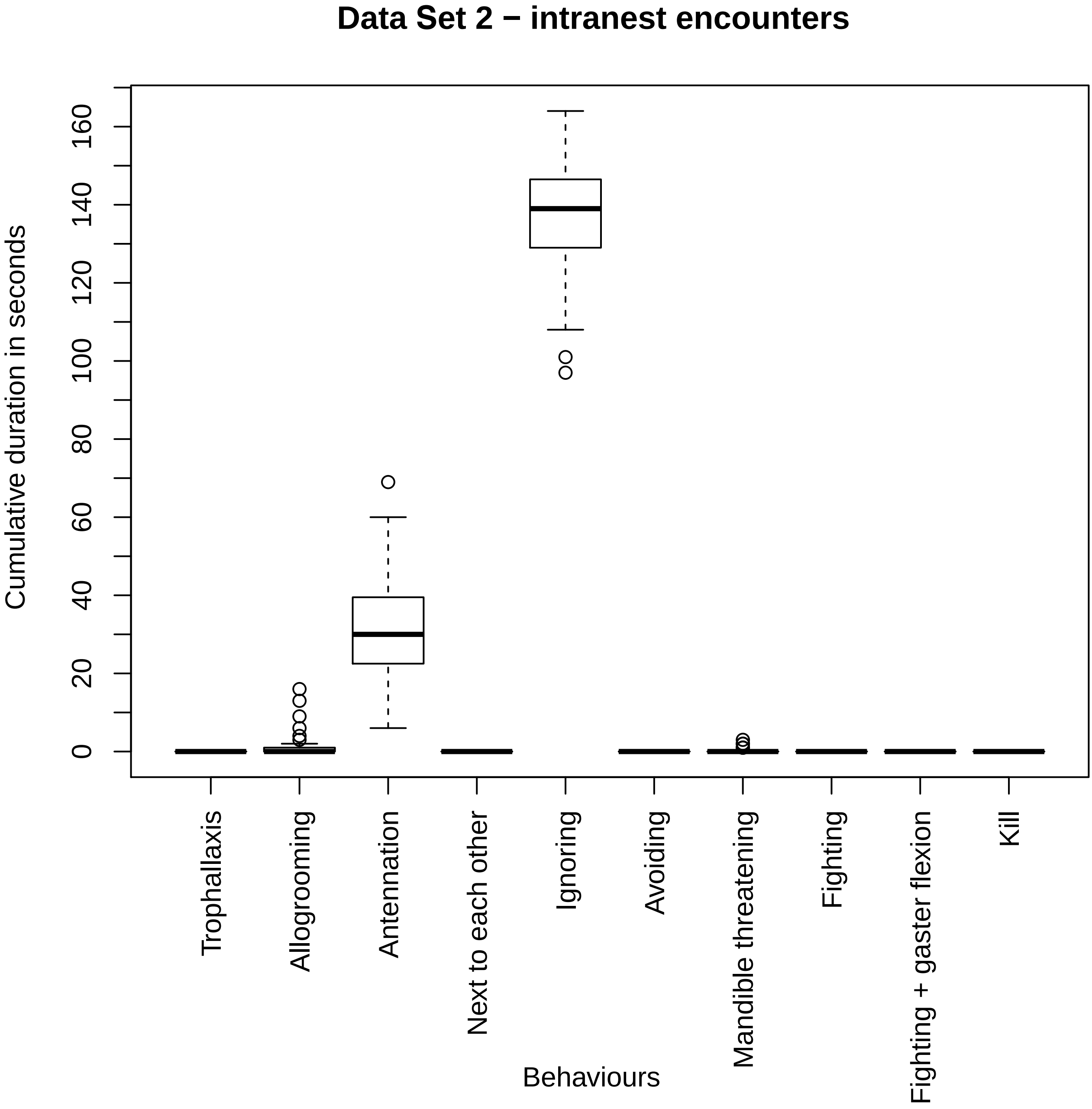

Fig. S4. Behaviour frequency boxplot of all intranest encounters of Data Set 2 (n = 6).

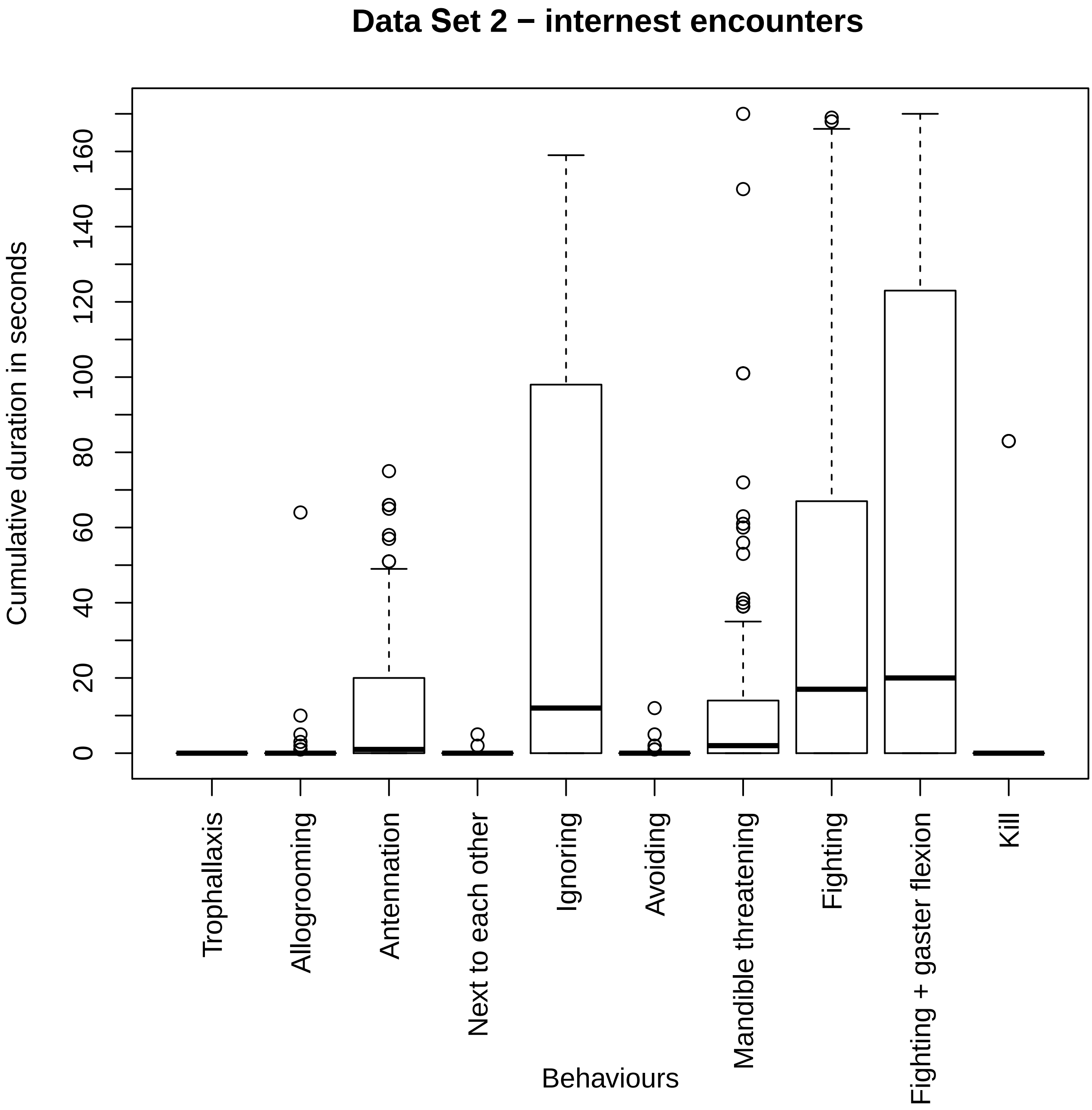

Fig. S5. Behaviour frequency boxplot of all internest encounters of Data Set 2 (n = 22).

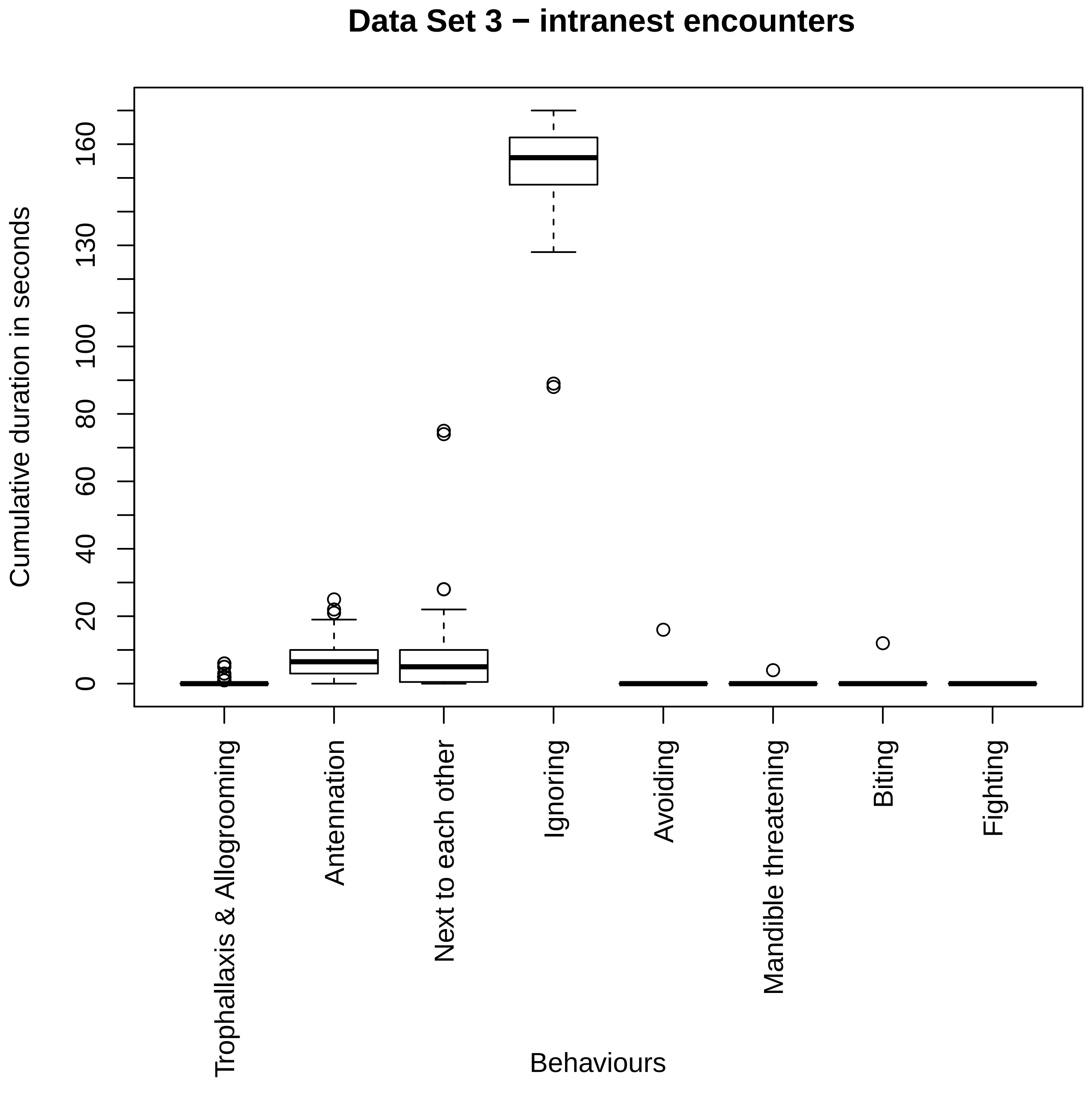

Fig. S6. Behaviour frequency boxplot of all intranest encounters of Data Set 3 (n = 11).

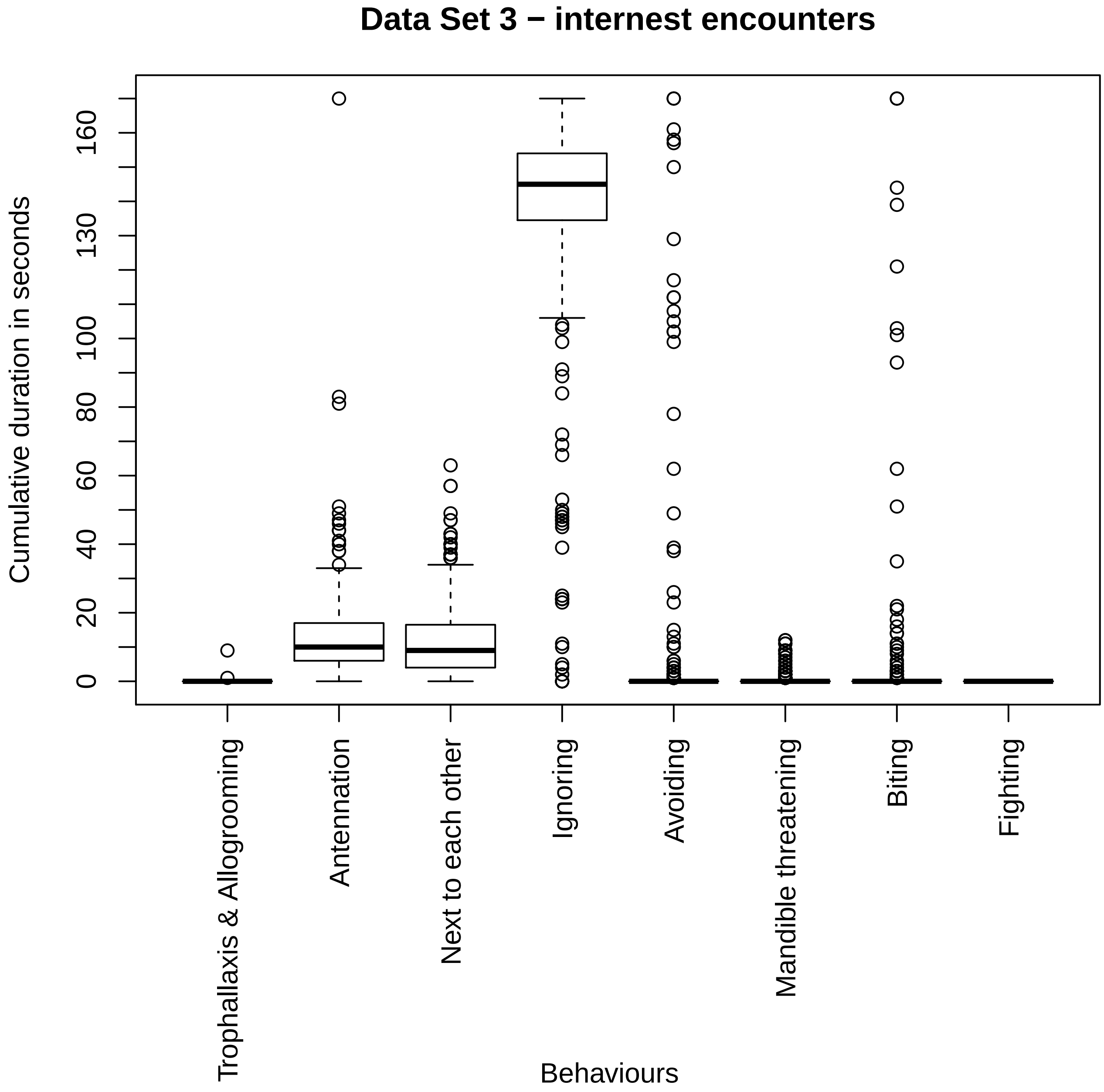

Fig. S7. Behaviour frequency boxplot of all internest encounters of Data Set 3 (n = 55).
